# Myocardial diffusion tensor imaging using diffusion-prepared SSFP

**DOI:** 10.1186/1532-429X-15-S1-P1

**Published:** 2013-01-30

**Authors:** Elizabeth M Tunnicliffe, Joseph Suttie, Rina Ariga, Stefan Neubauer, Matthew D Robson

**Affiliations:** 1 AVIC, Radcliffe Department of Medicine, University of Oxford, Oxford, UK; 2 OCMR, Radcliffe Department of Medicine, University of Oxford, Oxford, UK

## Background

Diffusion tensor imaging of the myocardium is challenging due to the large bulk motion of the heart relative to the distance water diffuses. One solution is to use diffusion gradients on two consecutive heartbeats, with EPI to readout the stimulated echo [1]. The technique has not been widely adopted, primarily due to the long imaging times required to overcome the low SNR of the technique. Recent new technology such as 3T scanners and 32-channel cardiac arrays improve the SNR, helping to make this approach feasible clinically. SSFP provides reduced distortion and high image quality, therefore we investigated the feasibility of replacing the EPI readout with SSFP for myocardial diffusion tensor imaging at 3T.

## Methods

**Figure 1:**
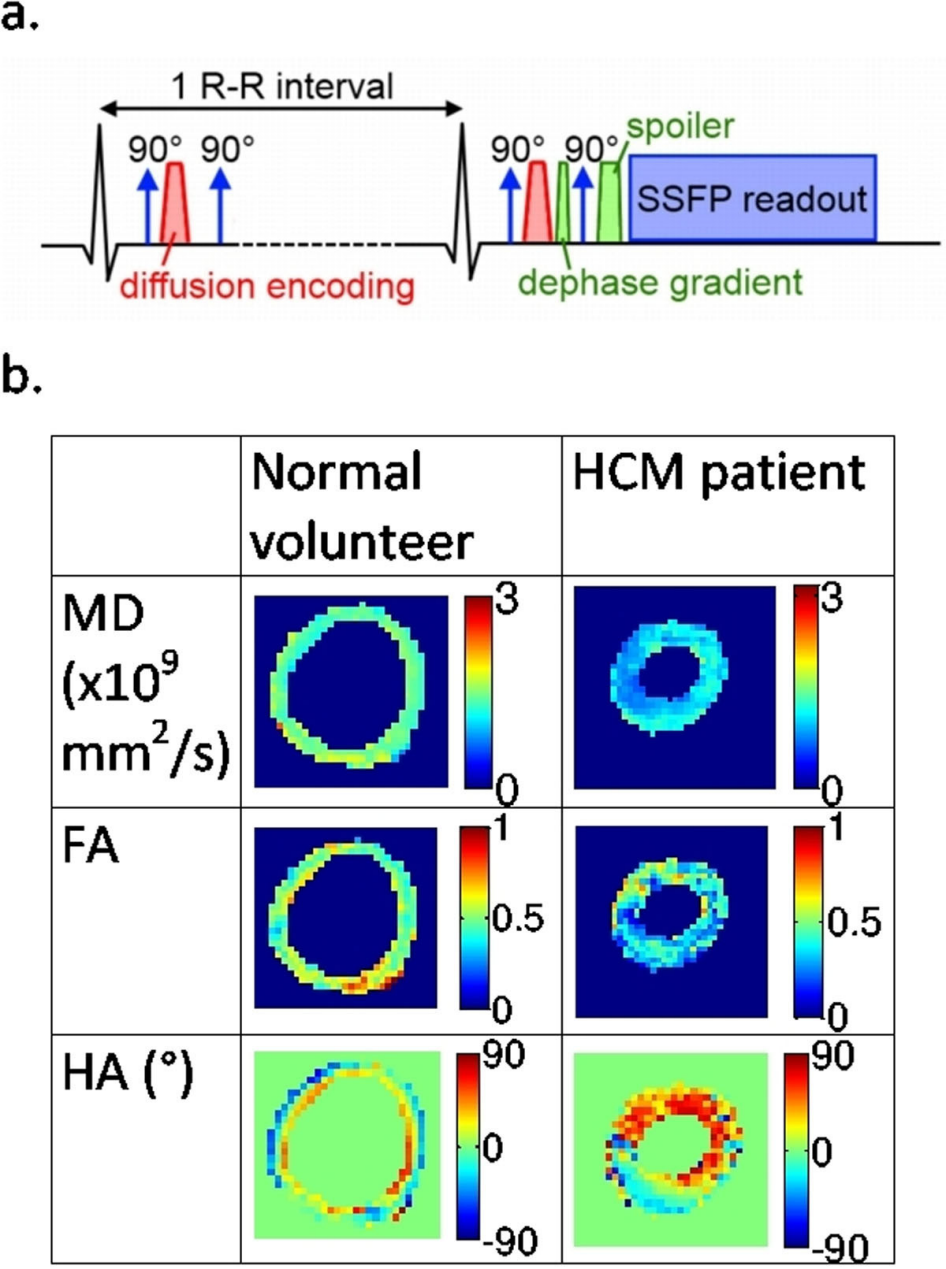
a. A schematic diagram of the diffusion-prepared SSFP sequence. b. Example maps from a normal volunteer and a hypertrophic cardiomyopathy patient.

The modified ECG-gated SSFP sequence including a diffusion preparation module is shown in Figure 1a. A final 90° tip-up pulse was required to enable an SSFP readout module rather than EPI. In order to avoid signal voids due to phase accrued from sub-millimetre bulk motion between the two diffusion gradients, a dephase gradient in the slice direction was included before the tip-up pulse, with this residual phase gradient rewound during each readout [2].

The sequence was tested on a 3T Siemens Trio using a 32-channel coil. Images were acquired in diastole (650 ms after the R-wave). Two heartbeats for T1 recovery were included between each imaging module, and the following readout parameters were used: TR/TE=2.5/1.3 ms,α=120°, bandwidth 1021 Hz/px, GRAPPA (R=2), matrix size 96x92, voxel size 2.7x2.7x10 mm. One b=0 image and 3 directions with prescribed b=300 s/mm^2^ were acquired in each 14-heartbeat breathhold, with three averages, requiring six breathholds for a single slice. Data were analysed in Matlab and mean diffusivity (MD), fractional anisotropy (FA) and helix angle (HA) calculated. A mid-ventricular slice was acquired in three normal volunteers and one patient with known hypertrophic cardiomyopathy (HCM).

## Results

Maps of MD, FA and HA for one normal volunteer and the HCM patient are shown in Figure 1b, demonstrating that this novel technique allows the acquisition of distortion-free images at 3T, and that the diffusion tensor and derived quantities can be calculated. The MD and FA for the three volunteers and one patient are reported in Table 1. FA shows good agreement with recently reported literature values [3], but the MD is slightly higher. This is due to T1-weighting of the signal due to the recovery of non-diffusion-weighted signal during the readout, which introduces a small positive bias to the MD [4].

A large change in FA is observed in the patient with HCM, as showed previously [5].

**Table 1:** Results for mean diffusivity and fractional anisotropy for three normal volunteers and one patient with HCM.

	Mean diffusivity (x10^-6^ mm^2^/s)	Fractional anisotropy
Normal volunteers (n=3)	1204±113	0.62±0.08
HCM patient (n=1)	1053	0.37

## Conclusions

We have demonstrated the feasibility of diffusion-prepared SSFP for diffusion tensor imaging of the myocardium at 3T, and shown its sensitivity to the presence of myocardial disarray in HCM.

## Funding

Part of this research was funded by the National Institute for Health Research (NIHR) Oxford Biomedical Research Centre based at The Oxford University Hospitals Trust at the University of Oxford. The views expressed are those of the author(s) and not necessarily those of the NHS, the NIHR or the Department of Health. We also acknowledge funding from the UK Department of Health.

## References

[B1] Edelman MRM 32 423 10.1002/mrm.1910320320 7984077

[B2] Lin MRM 60 8

[B3] Nielles-Vallespin MRM 2012 in print

[B4] Jeong MRM 50 821 10.1002/mrm.10593 14523969

[B5] Tseng JMRI 23 1

